# CpG dinucleotide methylation of the *SPDEF* gene as a blood-based epigenetic biomarker for prostate cancer diagnosis

**DOI:** 10.1186/s12894-025-01824-5

**Published:** 2025-06-02

**Authors:** Mohammad Vahid Ahmadianpour, Seyed Ahmad Aleyasin

**Affiliations:** Department of Medical Biotechnology, Medical Genetic Group, National Institute of Genetic Engineering and Biotechnology (NIGEB), Tehran, 14977-16316 Tehran Iran

**Keywords:** *SPDEF*, CpG methylation, Peripheral blood leukocytes, Prostate cancer, BPH, Total PSA (tPSA)

## Abstract

**Background:**

Prostate cancer (PCa) is the second most commonly diagnosed malignancy in men and is projected to cause approximately 35,250 deaths in 2024. The utility of total prostate-specific antigen (PSA) testing as a routine screening tool remains controversial due to limited specificity. Therefore, the identification of novel, noninvasive biomarkers is essential for improving early diagnosis. This study aimed to evaluate the methylation status of a specific CpG dinucleotide within the *SPDEF* (SAM-pointed domain-containing ETS transcription factor) gene promoter in blood leukocytes of PCa patients, using benign prostatic hyperplasia (BPH) samples as a control group.

**Methods:**

Peripheral blood samples were collected from 360 men, including 180 diagnosed with PCa and 180 with BPH. A target CpG dinucleotide (cg11346722) in the *SPDEF* promoter was selected based on analysis of The Cancer Genome Atlas (TCGA) data. Methylation levels were assessed using methylation-sensitive restriction enzyme PCR (MSRE-PCR) and quantitative PCR (qPCR). Associations between methylation and clinical parameters—tumor stage (TS), histological grade, and total PSA levels—were analyzed.

**Results:**

The mean methylation level at the *SPDEF* CpG site was significantly lower in PCa patients (hypomethylation: 92 ± 11.76%) compared to BPH controls (15.5 ± 15.12%) (*p* < 0.0001). Receiver operating characteristic (ROC) curve analysis demonstrated that *SPDEF* hypomethylation discriminated PCa from BPH with 98.3% sensitivity and 98.3% specificity at a < 55% methylation cutoff. A significant inverse correlation was observed between *SPDEF* methylation and both tumor stage (TS) and grade, whereas no correlation was found with total PSA levels.

**Conclusions:**

Hypomethylation of a specific CpG dinucleotide in the *SPDEF* promoter may serve as a promising noninvasive blood-based biomarker for the early detection and clinical stratification of prostate cancer.

**Graphical Abstract:**

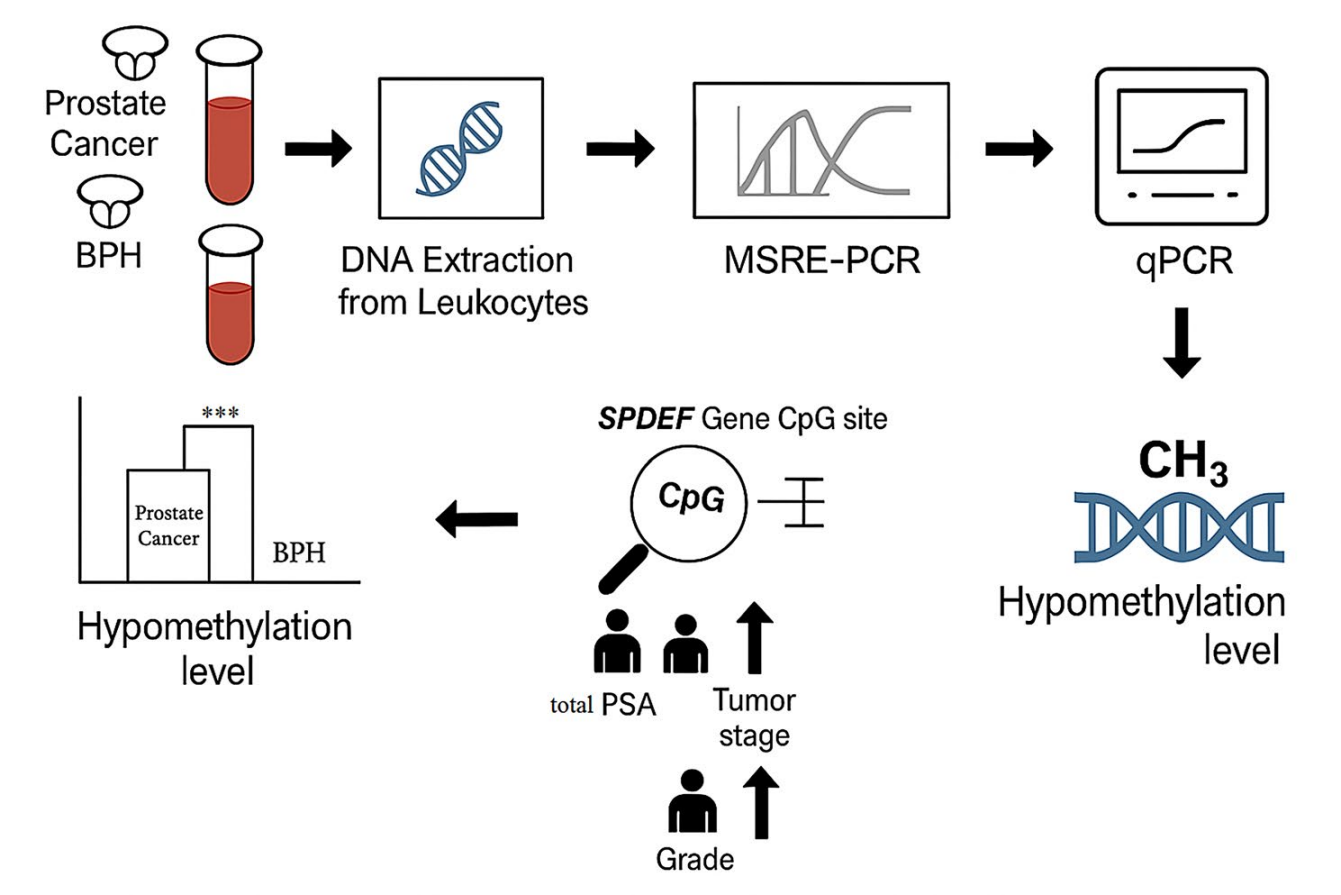

**Supplementary Information:**

The online version contains supplementary material available at 10.1186/s12894-025-01824-5.

## Introduction

Prostate cancer (PCa) is the fifth leading cause of cancer-related deaths globally, with approximately 1.27 million new cases reported each year. In the United States, PCa remains one of the most commonly diagnosed malignancies in men, with an estimated 299,010 new cases and 35,250 related deaths projected for 2024 [1]. Early-stage PCa often progresses slowly and remains asymptomatic, contributing to challenges in timely diagnosis [2, 3].

Currently, prostate-specific antigen (PSA) testing is the gold standard for PCa screening and early detection [4]. However, PSA lacks specificity, as elevated levels may also result from benign conditions such as benign prostatic hyperplasia (BPH), prostatitis, or increased prostate volume. These limitations have led the U.S. Preventive Services Task Force to recommend against routine PSA screening, underscoring the need for more accurate and specific biomarkers for PCa detection [4, 5].

PCa development and progression are closely linked to several clinical features, such as high Gleason scores and advanced tumor stage (TS) [6]. These features are significantly influenced by epigenetic mechanisms—particularly DNA hypomethylation—which occurs early in cancer and contributes to genomic instability, facilitating tumor initiation and progression through demethylation of CpG sites [6, 7].

Blood leukocytes (BLs) offer a convenient, non-invasive source for epigenetic biomarker discovery, and CpG methylation patterns in BLs have shown promise for the early diagnosis and prognosis of PCa [8, 9]. Previous studies have implicated hypomethylation of CpG sites in the LINE1 promoter in PCa disease [6].

The SAM-pointed domain-containing ETS transcription factor (SPDEF), located on chromosome 6p21.31 and comprising six exons [10, 11], belongs to the ETS transcription factor family. It was initially identified as *prostate-derived ETS factor (PDEF)* and is predominantly expressed in the luminal epithelial cells of the prostate. SPDEF directly interacts with the androgen receptor and functions as a coactivator, enhancing prostate-specific antigen (PSA) expression in LNCaP prostate cancer cells [12, 13]. Previous studies have implicated SPDEF in prostate cancer (PCa) progression and metastasis [14].

The methodology involved a comparative analysis of methylation levels at a specific CpG dinucleotide within the *SPDEF* promoter region (chr6:34544344–34544482) in DNA extracted from whole blood leukocytes of PCa patients and BPH controls. Using methylation-sensitive restriction enzyme PCR (MSRE-PCR) and quantitative PCR (qPCR), we assessed methylation alterations and their correlation with TS, Grade (ISUP), and serum total PSA levels.

This study aimed to evaluate the methylation status of a specific CpG dinucleotide *(*cg11346722*)* in the *SPDEF* gene promoter as a novel, cancer-specific epigenetic biomarker with both diagnostic and prognostic potential for prostate cancer. Using DNA extracted from whole blood leukocytes, the study addresses the need for improved diagnostic specificity while also supporting the utility of *SPDEF* methylation in disease monitoring and risk stratification.

## Materials and methods

### Study population and eligibility criteria

This study enrolled 360 men aged 43 years and older, recruited between January 2020 and December 2024 from the Urology Departments of Labbafinejad and Imam Khomeini Hospitals, Tehran, Iran. Among these, 180 had histologically confirmed prostate cancer (PCa), and 180 were diagnosed with benign prostatic hyperplasia (BPH), serving as the non-malignant control group.

Inclusion criteria for the PCa group:

- Elevated total PSA levels (\u22654 ng/mL).
- Abnormal digital rectal examination (DRE).
- Histopathological confirmation of PCa via transrectal ultrasound-guided biopsy (TRUS).

Exclusion criteria:

- Any prior oncologic treatment (e.g., surgery, radiotherapy, chemotherapy).
- Declined informed consent.

The BPH control group included patients with non-malignant prostatic enlargement, confirmed via clinical assessment, PSA levels (\u22651.5 ng/mL), DRE, and negative biopsy.

All participants provided written informed consent. The study was approved by the Ethics Committee of the National Institute of Genetic Engineering and Biotechnology (NIGEB), Tehran, Iran (Ethics Code: IR.A.2020.2.11). All procedures followed national regulations and the Declaration of Helsinki.

### Clinical assessment and laboratory measurements

For PCa patients, data collected included age, tumor stage (TS), and ISUP grade. Biopsies were performed under ultrasound guidance using a 12 + X-G needle. Tissue samples were taken from 6 peripheral and 6 transitional zone regions, with an additional core from any suspicious lesion [15].

BPH diagnosis was based on PSA levels, DRE, and histological confirmation. PSA levels were measured using a human kallikrein 3/PSA ELISA kit. Prostate volume was assessed by TRUS, and weight calculated using the elliptical formula [16]. Table 1 summarizes clinical and demographic variables. PSA and prostate weight showed significant differences between PCa and BPH (p \u2264 0.0001); age did not differ significantly (*p* = 0.658).

**Table 1:** Baseline characteristics of prostate cancer (PCa) and benign prostatic hyperplasia (BPH) patients included in the study (*n* = 180 per group). Values are presented as mean ± standard deviation (SD), median, and range (minimum–maximum). Total PSA (tPSA) levels were significantly higher in the PCa group (*p* ≤ 0.0001), while prostate weight was significantly higher in the BPH group (*p* ≤ 0.0001). No statistically significant difference was observed in age between groups (*p* = 0.658)

Baseline Parameters	*PCa (*n* = 180)	*BPH (*n* = 180)	*p*-Value
*Total PSA level (ng/mL)			≤0.0001
Mean ± SD	21.22 ± 21.33	6.3 ± 5.4	
Median	12	4.4	
Range	82	24.3	
Min-Max	1.3–82	0.4–24	
**Age (year)**			**≤0.6**
Mean ± SD	69.5 ± 17.67	68.65±15.6	
Median	69	70.5	
Range	50	52	
Min-Max	43–93	43–95	
**Weight_of_prostate (gr)**			**≤0.0001**
Mean ± SD	36.83 ± 38.36	56.26 ± 27.74	
Median	16	56	
Range	164	108.4	
Min–Max	4–168	1.6–110	

*Abbreviations: tPSA, total prostate-specific antigen; g, grams

### Whole-blood sampling and genomic DNA extraction

Following a minimum 12-hour overnight fast, 3 mL of peripheral whole blood was collected from each participant into EDTA-containing vacutainer tubes. Samples were obtained retrospectively from both prostate cancer (PCa) and benign prostatic hyperplasia (BPH) patients. For PCa patients, blood collection occurred after digital rectal examination (DRE), serum total PSA testing, and histological confirmation of cancer via prostate biopsy, but prior to any surgical or oncologic intervention, including prostatectomy, chemotherapy, or radiotherapy.

All clinical procedures mentioned—including PSA testing, DRE, and biopsy—were part of the standard diagnostic pathway routinely performed by urologists, and were not carried out specifically for this study. Participants were recruited after diagnosis had been established, and no additional clinical evaluations were conducted for research purposes.

Genomic DNA was extracted immediately after sample collection, using the GeneAll^®^ Exgene Blood SV Kit (GeneAll Biotechnology, Seoul, Korea), a rapid spin-column-based protocol that yields high-quality DNA in approximately 20 min. A total input of 3 mL whole blood was used per individual. Extracted DNA was stored at − 20 °C until further analysis.

DNA concentration was measured using a NanoDrop spectrophotometer (Thermo Scientific, Waltham, MA, USA). DNA quality and integrity were assessed by agarose gel electrophoresis, and purity was determined using the optical density ratio at 260 nm and 280 nm (A260/A280).

### Methylation analysis

Genomic DNA was isolated and subjected to methylation analysis at a target CpG site within the *SPDEF* promoter region (cg11346722). Two complementary PCR-based methods were employed:

- MSRE-PCR (Methylation-Sensitive Restriction Enzyme PCR): Genomic DNA was digested with the HaeIII enzyme, which selectively cuts unmethylated sites. Amplification was performed using primers flanking the target site, and digestion efficiency was assessed via agarose gel electrophoresis.
- Quantitative PCR (qPCR): The same primers were used in qPCR to quantitatively assess methylation. ΔCt values were obtained relative to undigested controls, and percentage methylation was calculated using the 2^–ΔCt method.

All samples were analyzed in duplicate for both assays, and data were used for comparative and statistical analyses.

### Identification of DMRs in the GEO datasets

Publicly available DNA methylation microarray datasets, obtained from the NCBI Gene Expression Omnibus (GEO) database (https://www.ncbi.nlm.nih.gov/geo/geo2r/*)*, were analyzed to identify differentially methylated regions (DMRs) in prostate cancer:

- GSE26126: Includes 94 tumor and 86 normal prostate tissue samples.
- GSE15727: Includes 23 tumor and 10 BPH samples.

Differential methylation analysis was conducted using beta-values, and log2 fold-change (_log2_FC) values were calculated. Adjusted p-values were determined using the Benjamini–Hochberg method to control the false discovery rate (FDR) in the context of multiple comparisons [17].

To identify shared hypomethylated genes, we filtered for genes with values of − 1 < log₂FC ≤ 0. The overlap between the two datasets was assessed using the online Venn diagram tool provided by the Bioinformatics and Systems Biology group (http://bioinformatics.psb.ugent.be/webtools/Venn/*).*

### Bioinformatic selection and primer design for CpG-specific MSRE-PCR targeting promoter hypomethylation

Promoter and regulatory regions of the target gene were identified using the GeneCards database (https://www.genecards.org*)*, focusing on regulatory elements associated with transcriptional control. The specific location of differentially methylated regions (DMRs) was determined using the SMART App (http://www.bioinfo-zs.com/smart/*)*, which provides methylation profiles across 33 cancer types using data from The Cancer Genome Atlas (TCGA) [18].

The UCSC Genome Browser (GRCh38/hg38; http://genome.ucsc.edu*)* was used to visually inspect the *SPDEF* gene locus and promoter region of interest (chr6:34544344–34544482). CpG dinucleotides within the promoter were predicted using the EMBOSS CpGPlot/CpGReport tool (https://www.ebi.ac.uk/Tools/seqstats/emboss_cpgplot*)*, applying standard thresholds for the observed-to-expected CpG dinucleotide ratio (Obs/Exp > 0.6) and GC content (> 50%) to identify regions likely to be involved in methylation regulation.

To identify suitable restriction enzyme recognition sites (not “islands”), the NEBcutter V2.0 tool (http://nc2.neb.com/NEBcutter2*)* was employed. Methylation-sensitive restriction enzymes (MSREs) were selected based on the presence of CpG dinucleotides within their recognition sequences.

To ensure that the methylation-sensitive restriction enzyme HaeIII was not inhibited and performed effective digestion, multiple quality control measures were implemented:

- Positive digestion control: DNA from samples previously shown to be unmethylated at the *SPDEF* promoter (cg11346722) was used to verify HaeIII activity. Complete digestion in these samples was evidenced by a significant reduction or complete absence of PCR amplification.
- Matched undigested controls: For each sample, an undigested aliquot (enzyme-free) served as a 100% methylated reference, enabling comparison of amplification levels.
- Reproducibility: All digestion reactions were conducted in at least duplicate. Replicates showed high concordance in band intensity (MSRE-PCR) and ΔCt values (qPCR), confirming the reliability of digestion outcomes.
- Optimal conditions: Digestions were carried out under the manufacturer-recommended conditions (2–3 h at 37 °C) using the appropriate buffer, followed by heat inactivation.

**Fig. 1:**
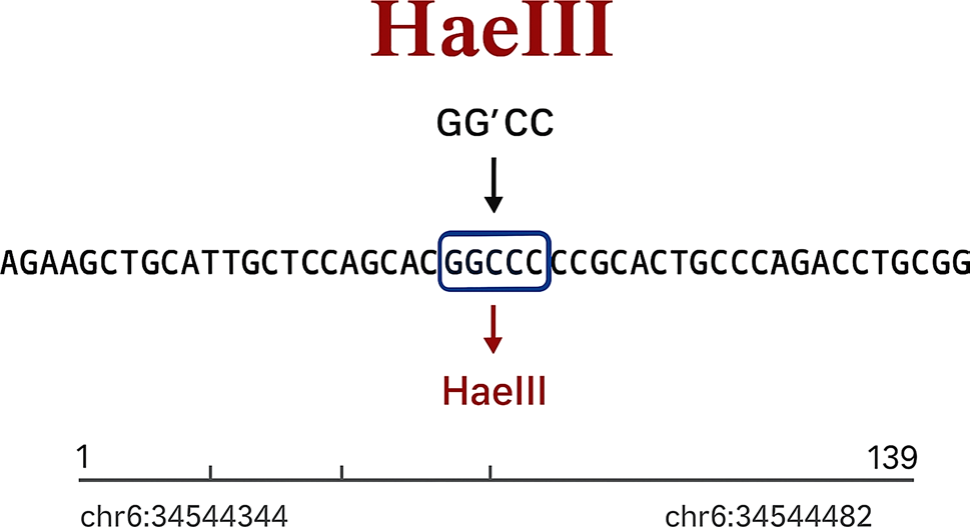
Visualization of the HaeIII restriction site at the *cg11346722* locus, located on chromosome 6 (chr6:34544344–34544482). The recognition sequence GG^CC is highlighted within the *SPDEF* promoter region, and the cleavage site of HaeIII is indicated by an arrow. This illustration is based on data from NEBcutter V2.0 (https://nc2.neb.com/NEBcutter2*)*, which was used to identify methylation-sensitive restriction enzyme (MSRE) recognition islands relevant to the design of the MSRE-PCR assay

This multipoint quality control strategy ensured specific and effective digestion of unmethylated CpG sites and validated the robustness of the methylation assay used in this study. A schematic overview of the promoter region and enzyme recognition sites is presented in Fig. 1.

Primers for MSRE-PCR were designed flanking the selected MSRE recognition site using Primer3Plus (https://primer3plus.com/). Primer characteristics—including melting temperature, GC content, amplicon size, and specificity—were optimized for MSRE-PCR conditions. Final primer sequences and design parameters are provided in Supplementary S2_Table-S3.

### CpG dinucleotide methylation analysis using MSRE-PCR

Methylation-sensitive restriction enzyme PCR (MSRE-PCR) is a rapid and sensitive method for detecting DNA methylation by comparing digested and undigested DNA samples. It utilizes methylation-sensitive restriction enzymes that selectively cleave unmethylated CpG sites, followed by PCR amplification with gene-specific primers. This approach enables detection of even low-abundance methylated DNA without requiring bisulfite conversion [19, 20].

In contrast, alternative methods such as pyrosequencing are limited by short read lengths, difficulties in analyzing short sequences, and susceptibility to equipment-related run failures [21]. Bisulfite sequencing, while widely used, involves harsh chemical treatment that can degrade up to 90% of DNA, reducing yield and reliability [22, 23].

In this study, MSRE-PCR was applied to evaluate methylation at the *SPDEF* CpG dinucleotide (cg11346722) located in the promoter region. The restriction enzyme HaeIII (recognition site: 5′-GG^CC-3′; Takara, Shiga, Japan) was used to digest unmethylated DNA. Reactions were conducted in the manufacturer’s recommended buffer at 37 °C for 3 h, followed by enzyme inactivation at 95 °C for 10 min.

To verify enzyme activity and prevent inhibition, parallel reactions with previously characterized unmethylated DNA were included. Complete digestion was confirmed by a marked reduction or absence of PCR amplification. This validation was performed in quadruplicate.

Each 10 µL digestion reaction included 50 ng of genomic DNA, 0.5 µL of HaeIII, 1 µL of 10× buffer, and nuclease-free water. A non-digested control was prepared alongside.

PCR amplification of digested and undigested DNA was performed using 10 µL of 2× Taq PCR Master Mix (Ampliqon, Denmark), 1 µL of digestion product, 0.5 µL each of forward (5′-GACCCACTCGACGTATCTCT-3′) and reverse (5′-ACCTCAGACCACAGGCAGGC-3′) primers (10 pmol/µL), and nuclease-free water to a final volume of 20 µL. The amplicon size was 176 bp. Additional MSRE-PCR conditions, enzyme details, and recognition sequences are provided in Supplementary S2 _Table-S1.

PCR products were resolved on 1.5% agarose gels stained with ethidium bromide. Band intensities were analyzed using GelAnalyzer 23.1 software.

To minimize pipetting variability, all samples were processed in quadruplicate. Band intensities were normalized against undigested controls run on the same gel. All pipetting was performed using calibrated micropipettes and low-retention tips by a trained operator.

Band intensity ratios (digested/undigested) were calculated to determine the degree of hypomethylation. These results were subsequently validated by qPCR analysis (see Sect. 2.8).

### Quantification of methylation status using quantitative PCR (qPCR)

The MSRE-PCR assay described above provided a semi-quantitative assessment of *SPDEF* methylation. However, to account for potential variability due to gel resolution or pipetting inconsistencies, quantitative PCR (qPCR) was also performed to more precisely quantify methylation levels.

qPCR amplification was conducted on both digested and undigested DNA samples using a Rotor-Gene 6000 thermal cycler. The thermal cycling parameters and reaction conditions are listed in Supplementary S2 _Table-S1.

Methylation percentage was calculated using a ΔCt-based formula:

% Methylation = 100 × e^(–0.7 × ΔCt).

where ΔCt = Ct(digested)– Ct(undigested), and Ct is the cycle number at which fluorescence surpasses the detection threshold.

This formula assumes an amplification efficiency of approximately 100% and is based on the principle that methylated DNA remains intact after restriction enzyme digestion and is more efficiently amplified than unmethylated DNA, which is cleaved and thus yields a higher Ct value. A larger ΔCt indicates a greater degree of digestion and, therefore, a lower degree of methylation.

The exponential coefficient − 0.7 was empirically optimized in preliminary experiments and is consistent with coefficients reported in similar MSRE-qPCR workflows [24]. It reflects the relationship between DNA template concentration, enzymatic digestion efficiency, and amplification dynamics.

To validate this quantification method, a standard curve was established using commercially available control DNA at defined methylation levels (0%, 25%, 50%, 75%, and 100%), prepared by mixing fully methylated and unmethylated genomic DNA. These standards were processed under identical digestion and qPCR conditions as test samples. The resulting Ct values were plotted against log-transformed methylation percentages to construct a calibration curve, which demonstrated strong linear correlation (R² > 0.98). This confirmed the reproducibility and accuracy of the ΔCt-based formula and the appropriateness of the − 0.7 coefficient for this assay.

### Sample grouping based on methylation levels

**Fig. 2:**
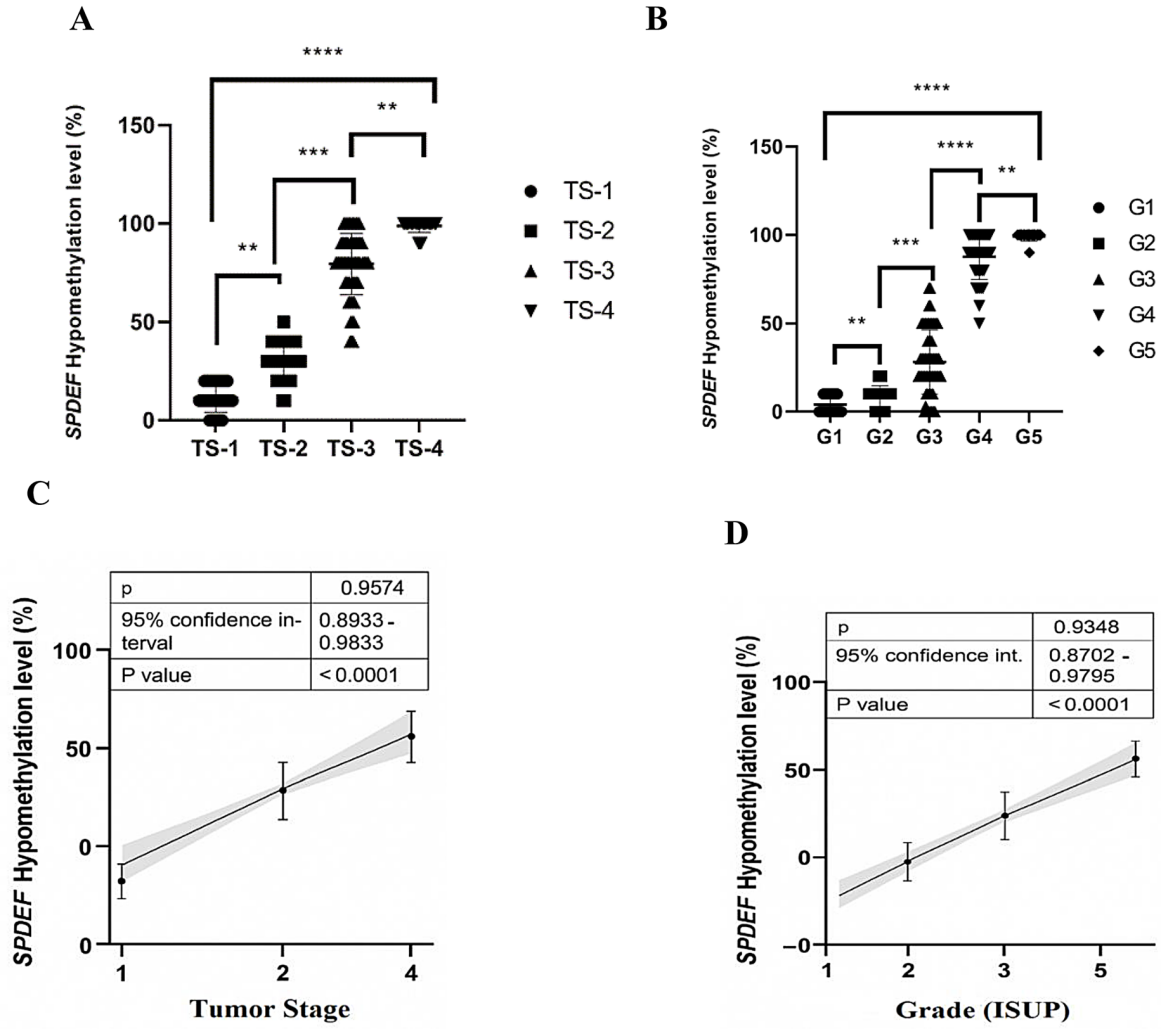
Correlation of *SPDEF* promoter hypomethylation with tumor stage and histological grade in prostate cancer. **A**,** B-**Group-wise comparison of *SPDEF* CpG hypomethylation levels across tumor stage (TS1–TS4) and ISUP grade groups (G1–G5) using the Kruskal–Wallis test followed by Dunn’s multiple comparisons. Hypomethylation levels increased progressively with advancing stage and grade. **C**,** D-**Linear regression analysis confirmed strong positive correlations between SPDEF hypomethylation and both tumor stage (*r* = 0.9574) and ISUP grade (*r* = 0.9348), with tight 95% confidence intervals and highly significant *P* values (< 0.0001). These results support the potential of SPDEF hypomethylation as a biomarker of prostate cancer progression

For comparative analysis of *SPDEF* CpG dinucleotide hypomethylation with clinicopathological parameters such as tumor stage (TS), ISUP grade, and PSA subgroups, patient samples were stratified into groups according to their assigned clinical classifications. Hypomethylation levels were not used to pre-group samples. Instead, hypomethylation data were analyzed post hoc within each predefined clinical group to assess statistical associations. For correlation and comparison analysis, non-parametric tests (e.g., Kruskal–Wallis with Dunn’s multiple comparisons, and Mann–Whitney U test) were employed due to the ordinal nature and non-Gaussian distribution of clinicopathological variables (Fig. 2A-D).

### The assessment of the relationship between PSA levels and *SPDEF* promoter methylation

**Fig. 3:**
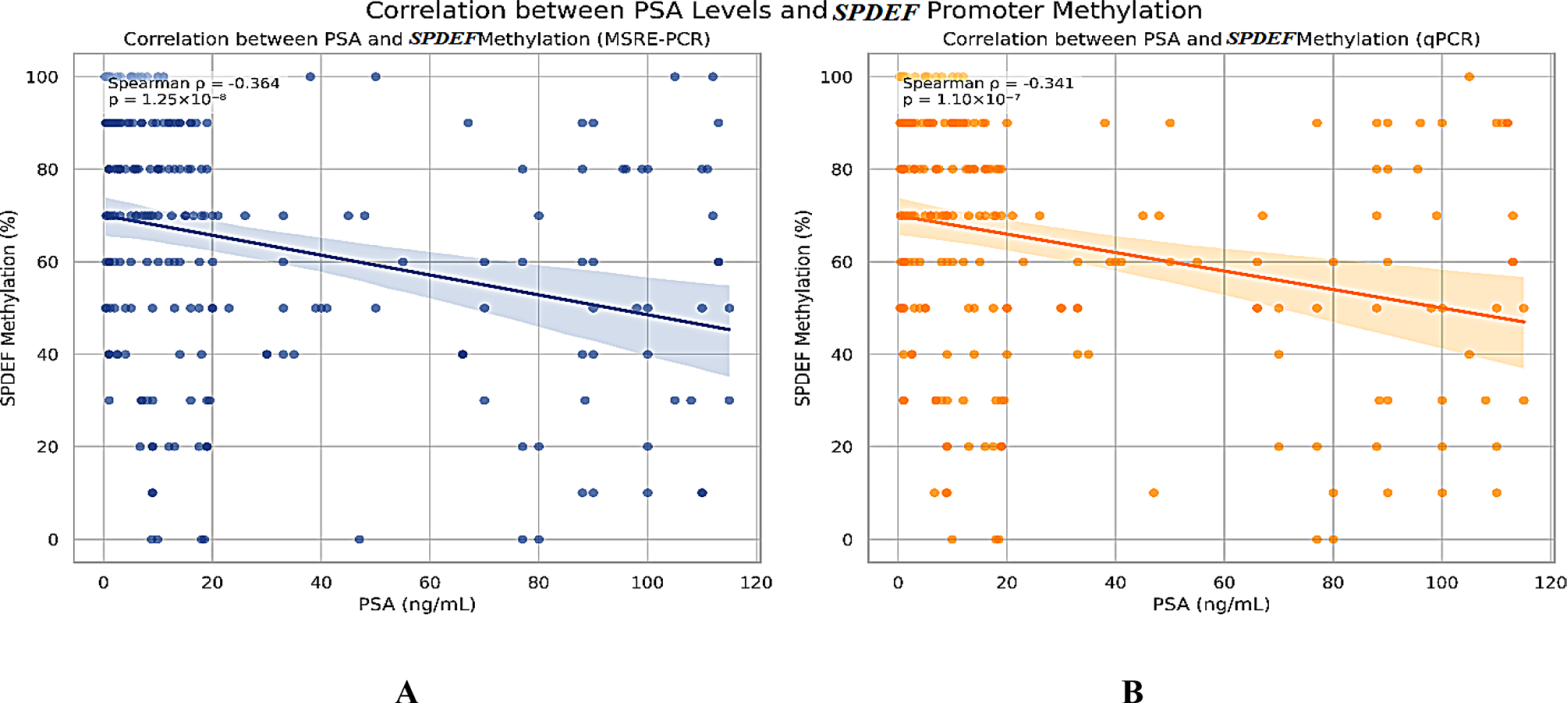
Correlation between serum PSA levels and *SPDEF* promoter methylation measured by two PCR-based methods. Scatter plots show the relationship between serum PSA levels (ng/mL) and *SPDEF* promoter methylation percentages as quantified by (**A**) methylation-sensitive restriction enzyme PCR (MSRE-PCR) and (**B**) quantitative PCR (qPCR). Trendlines represent linear regression for visual interpretation. Spearman’s rank correlation coefficient (ρ) and associated *p*-values are displayed on each panel. Both methods revealed a moderate, statistically significant inverse correlation, indicating that higher PSA levels are associated with decreased methylation of the *SPDEF* promoter

To assess the relationship between *SPDEF* promoter methylation and serum PSA levels, PSA values were treated as a continuous variable. Spearman’s rank correlation coefficient was used to evaluate the association between PSA levels and methylation percentages obtained via both MSRE-PCR and qPCR assays, given the non-normal distribution of clinical variables and the ordinal nature of PSA progression. This approach avoided subdivision of the diagnostic gray zone (4–10 ng/mL), in line with the reviewer’s recommendations, and provided a more robust statistical analysis (Fig. 3).

### In Silico analysis of *SPDEF* expression and immune cell infiltration in prostate cancer

To explore the potential relationship between *SPDEF* gene expression and immune cell infiltration in prostate cancer, we conducted an in silico analysis using the TIMER2.0 database (http://timer.cistrome.org/).

TIMER2.0 provides immune infiltration estimates based on gene expression data from TCGA datasets. We utilized the single-sample Gene Set Enrichment Analysis (ssGSEA) algorithm implemented in TIMER2.0 to assess correlations between *SPDEF* expression levels and infiltration of various immune cell types, including CD8⁺ T cells, in prostate adenocarcinoma (PRAD) samples. This analysis was exclusively computational and based on publicly available transcriptomic data. No experimental (wet-lab) validation was performed in this study. Future work may include biological validation of these associations to further elucidate the immunoregulatory role of SPDEF in the tumor microenvironment.

### Analysis of statistical data

All statistical analyses were performed using GraphPad Prism version 9.0.0 (GraphPad Software, La Jolla, CA, USA) and IBM SPSS Statistics version 26 (IBM Corp., Armonk, NY, USA). Data are presented as mean ± standard deviation (SD), unless otherwise specified.

Group-wise comparisons of *SPDEF* hypomethylation levels across tumor stages (TS1–TS4) and ISUP grades (G1–G5) were conducted using the Kruskal–Wallis test, followed by Dunn’s multiple comparisons to determine statistically significant differences between groups.

Spearman’s rank correlation coefficient was used to assess the relationship between SPDEF hypomethylation and ordinal clinicopathological parameters, including tumor stage and ISUP grade. Pearson’s correlation was applied only when evaluating relationships between continuous variables such as PSA levels and *SPDEF* methylation levels.

Receiver operating characteristic (ROC) curve analysis was conducted in GraphPad Prism to evaluate the diagnostic accuracy of *SPDEF* promoter methylation status in distinguishing prostate cancer from BPH. Sensitivity, specificity, and area under the curve (AUC) were calculated.

A p-value of < 0.05 was considered statistically significant in all tests.

## Results

### Identification and prioritization of *SPDEF* as a candidate biomarker

To explore potential epigenetic biomarkers for prostate cancer (PCa), we performed in silico screening using three publicly available datasets: GSE26126, GSE15727, and TCGA-PRAD. Differential methylation analysis via GEO2R identified thousands of significantly altered CpG sites between tumor and non-tumor tissues.

**Fig. 4:**
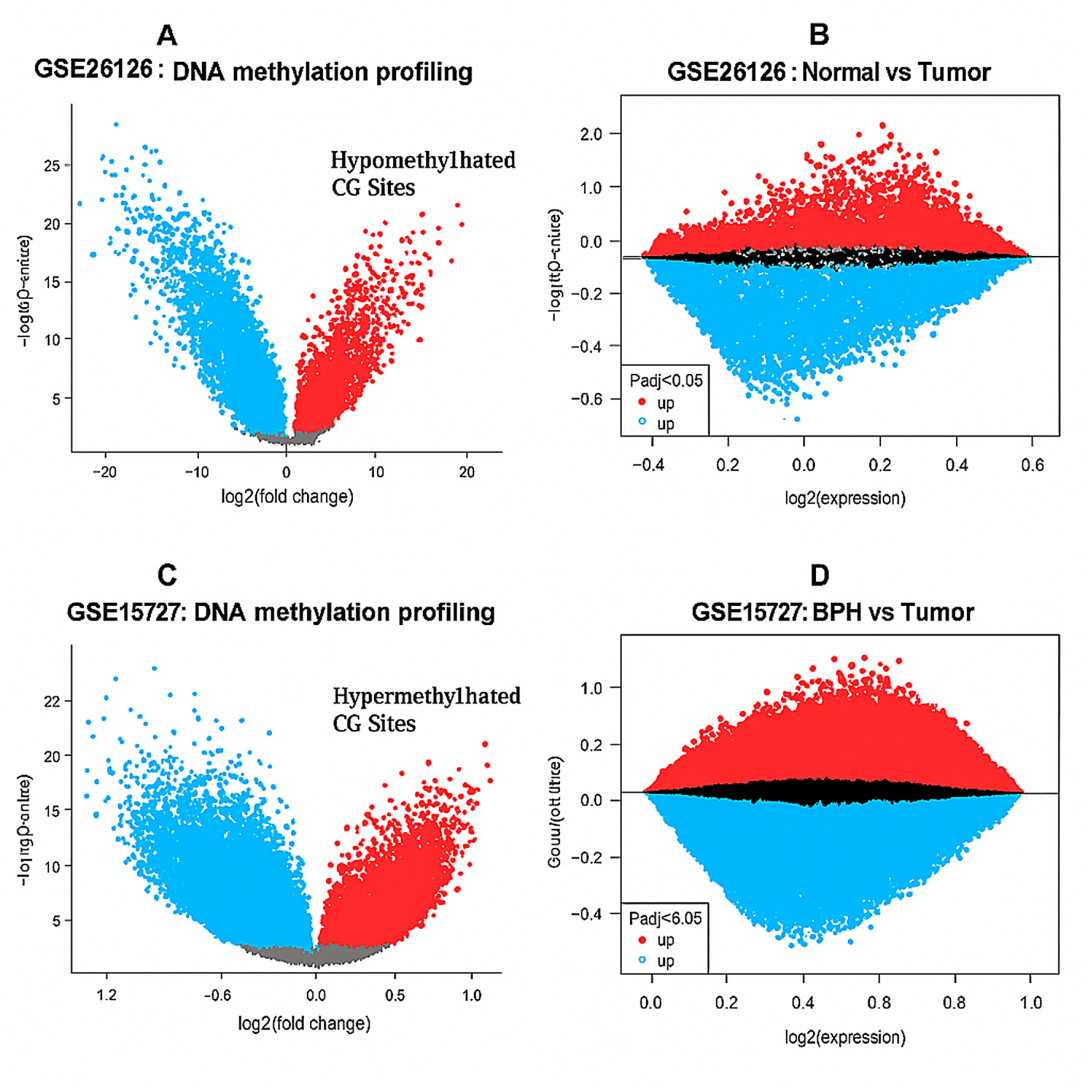
Genome-wide methylation and gene expression profiling identifies *SPDEF* as a candidate biomarker in prostate cancer. Volcano plots illustrating differential DNA methylation (left panels) and gene expression (right panels) in prostate cancer tissues compared to non-malignant controls from two GEO datasets. **A**–**B**: Dataset GSE26126 includes 94 tumor and 86 normal prostate samples. Red dots represent hypomethylated CpG sites or upregulated genes; blue dots indicate hypermethylation or downregulation. **C**–**D**: Dataset GSE15727 includes 23 tumor and 10 BPH samples. Same color scheme is used for visualization. *SPDEF* was identified among the most significantly hypomethylated genes in both datasets. A specific CpG site, cg11346722, located within the *SPDEF* promoter, showed consistent hypomethylation and was selected for downstream validation in blood-derived DNA using MSRE-PCR and qPCR. This selection was based on statistical significance (*p* < 0.05), consistent fold-change, and genomic annotation (promoter and transcription factor binding overlap)

To narrow the search, we filtered for hypomethylated targets (− 1 < log₂FC ≤ 0) and used a Venn diagram analysis to identify shared features. A total of 591 genes were consistently hypomethylated across the two GEO datasets (Fig. 4A–D; Supplementary S3_Fig.1; Supplementary File S3_Geneslist.xlsx).

**Fig. 5:**
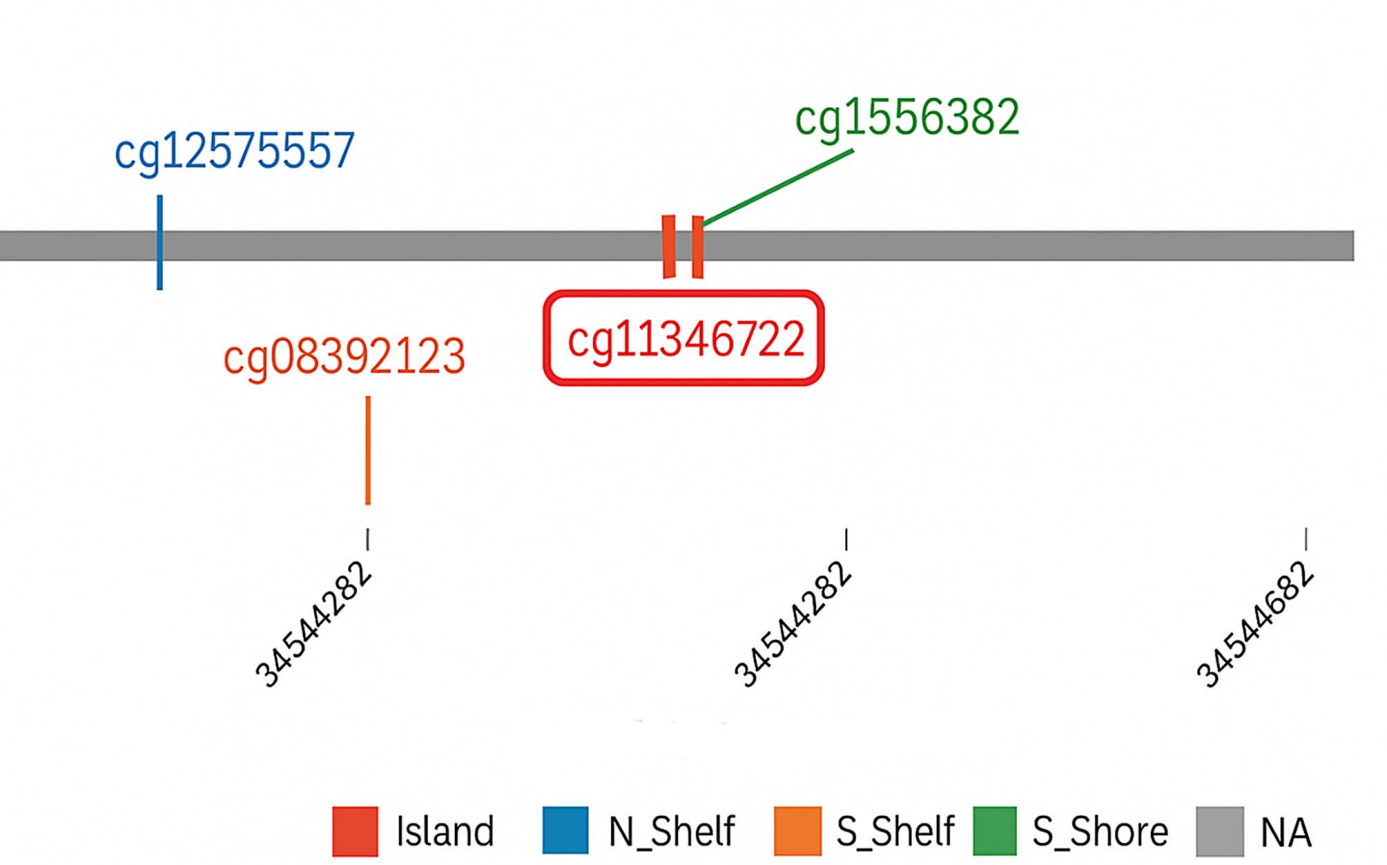
Genomic context and CpG site classification of cg11346722 on chromosome 6. The figure displays the relative positions of CpG sites cg12575557, cg08392123, cg11346722, cg15658328, and cg15954235 across the *SPDEF* promoter region (chr6:34544344–34544882). The cg11346722 site—highlighted in red—is located within a CpG island, outlined in dark red. Other CpG sites are distributed across various genomic regions, including the North Shelf (N_Shelf), South Shelf (S_Shelf), and South Shore (S_Shore), as indicated by their respective color codes. The grey bar represents non-annotated regions (NA). This genomic map was visualized using the UCSC Genome Browser and annotation tools (http://www.bioinfozs.com/smart-app), illustrating the distribution of CpG sites within the promoter regulatory region of the *SPDEF* gene.

Among these, *SPDEF* emerged as a robust candidate with statistically significant hypomethylation (adjusted *p* < 0.05) in both GSE26126 and GSE15727 (Supplementary S3 Fig. 5). Notably, the CpG site cg11346722, located within the *SPDEF* promoter region (chr6:34544344–34544482), exhibited reproducible tumor-specific hypomethylation and overlapped with functionally annotated regulatory elements, including promoter/enhancer regions and transcription factor binding sites (UCSC/ENCODE tracks).

Given its consistent bioinformatic profile and genomic context, cg11346722 was prioritized for further validation. We designed a dual-assay approach using methylation-sensitive restriction enzyme PCR (MSRE-PCR) for semi-quantitative analysis and qPCR for quantitative assessment. This methodological redundancy provided robust confirmation of cg11346722 methylation levels in our clinical cohort.

### Validation of *SPDEF* methylation and expression using TCGA and GEO datasets

**Fig. 6:**
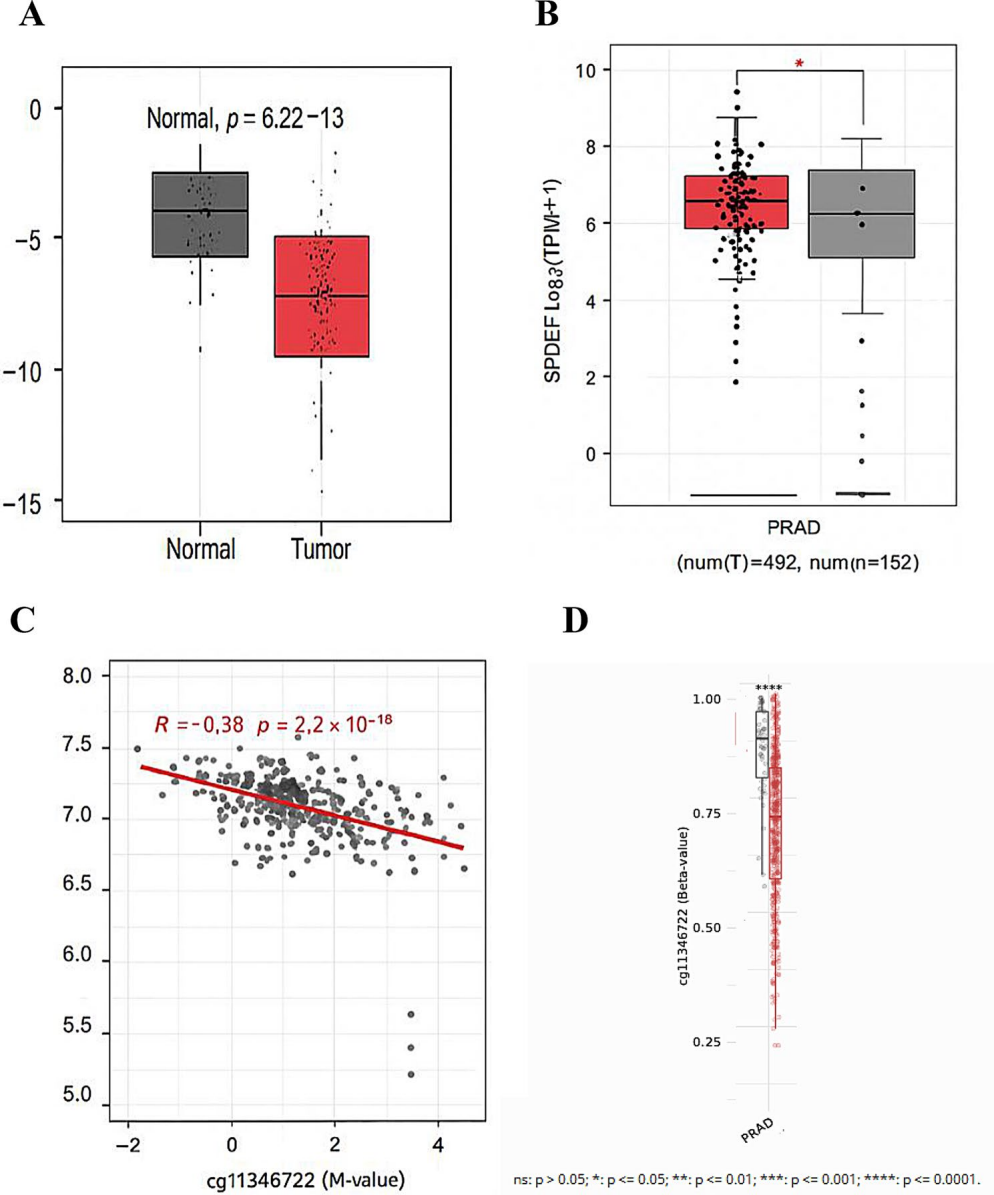
Integrated analysis of *SPDEF* gene methylation and expression in prostate adenocarcinoma (PRAD) and normal prostate tissues. A**-**Boxplot comparing DNA methylation levels (beta values) at the cg11346722 CpG dinucleotide within the *SPDEF* promoter region (Chr6:34544344–34544482) between normal (gray) and tumor (red) prostate tissue samples. A significant reduction in methylation was observed in tumor samples (Wilcoxon *p* = 6.2 × 10⁻¹³), indicating *SPDEF* promoter hypomethylation in PRAD. **B-**Boxplot showing *SPDEF* gene expression levels (log₂[TPM + 1]) in PRAD tumor samples (*n* = 492, red) versus normal tissues (*n* = 152, gray). Tumor tissues exhibited significantly higher *SPDEF* expression (*p* < 0.05; red asterisk). This inverse pattern supports epigenetic regulation of *SPDEF* via DNA methylation. Data obtained from GEPIA2 (http://gepia2.cancer-pku.cn*).***C-**Scatterplot showing a significant inverse correlation between methylation M-values at cg11346722 and *SPDEF* expression (log₂[TPM + 1]) across 76 PRAD samples (*R* = − 0.38, *p* < 2.2 × 10⁻¹⁶), indicating that increased methylation at this CpG dinucleotide is associated with decreased *SPDEF* transcription. Data obtained from SMART App(http://www.bioinfo-zs.com/smartapp*).***D-**Independent dataset confirming reduced methylation (beta values) at cg11346722 in PRAD tumor tissues compared to normal samples. Each dot represents an individual sample. The consistent hypomethylation observed in tumors (*p* < 0.0001) reinforces the potential of cg11346722 as an epigenetic biomarker in prostate cancer

To validate the association between *SPDEF* promoter methylation and gene expression in prostate cancer, we analyzed data from the TCGA PRAD cohort and publicly available GEO datasets. Methylation levels at the CpG dinucleotide cg11346722, located within the *SPDEF* gene region (Chr6:34544344–34544482), were significantly lower in tumor samples compared to normal prostate tissues (Wilcoxon *p* = 6.2 × 10⁻¹³; Fig. 6A). This hypomethylation was accompanied by a significant increase in *SPDEF* mRNA expression in tumor tissues (*p* < 0.001; Fig. 6B).Regression analysis revealed a statistically significant inverse correlation between methylation at cg11346722 and *SPDEF* expression (*R* = − 0.38, *p* < 2.2 × 10⁻¹^6^), supporting the link between reduced methylation and transcriptional activation (Fig. 6C). These findings were further corroborated by an independent dataset, which also demonstrated a consistent reduction in methylation at cg11346722 in tumor samples (Fig. 6D; Supplementary S4 _Figs. S1 and S2).Together, these results suggest that hypomethylation at the specific CpG site cg11346722 may serve as a regulatory mechanism contributing to the upregulation of *SPDEF* in prostate cancer.

### Comparison and validation of *SPDEF* methylation by MSRE-PCR and qPCR

**Fig. 7:**
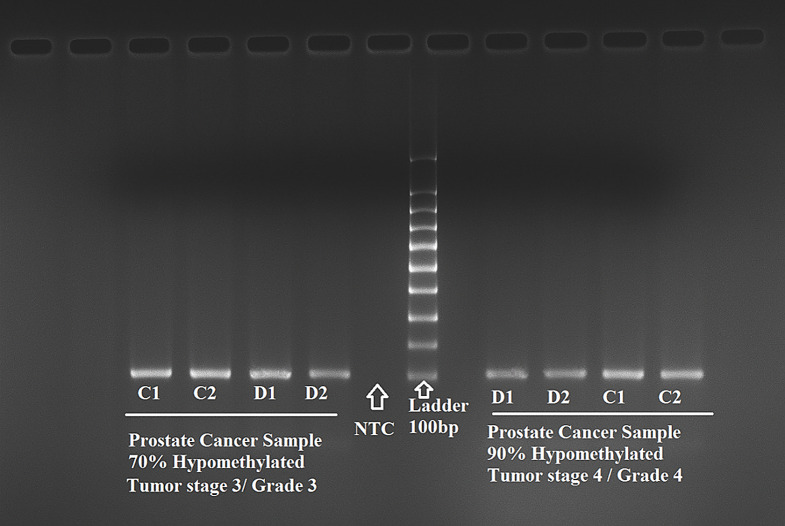
Methylation-sensitive restriction enzyme PCR (MSRE-PCR) gel electrophoresis image showing differential methylation of the *SPDEF* promoter (cg11346722) in representative clinical prostate cancer samples (*n* = 360; 180 PCa and 180 BPH)

To validate the bioinformatic findings from the GEO datasets (GSE26126 and GSE15727), we analyzed DNA from 360 clinical blood samples (180 PCa and 180 BPH). The methylation status of the *SPDEF* CpG dinucleotide (cg11346722) was first assessed using methylation-sensitive restriction enzyme PCR (MSRE-PCR). Figure 7 shows a representative gel image, where band intensities were evaluated in both PCa and BPH groups.

**Fig. 8:**
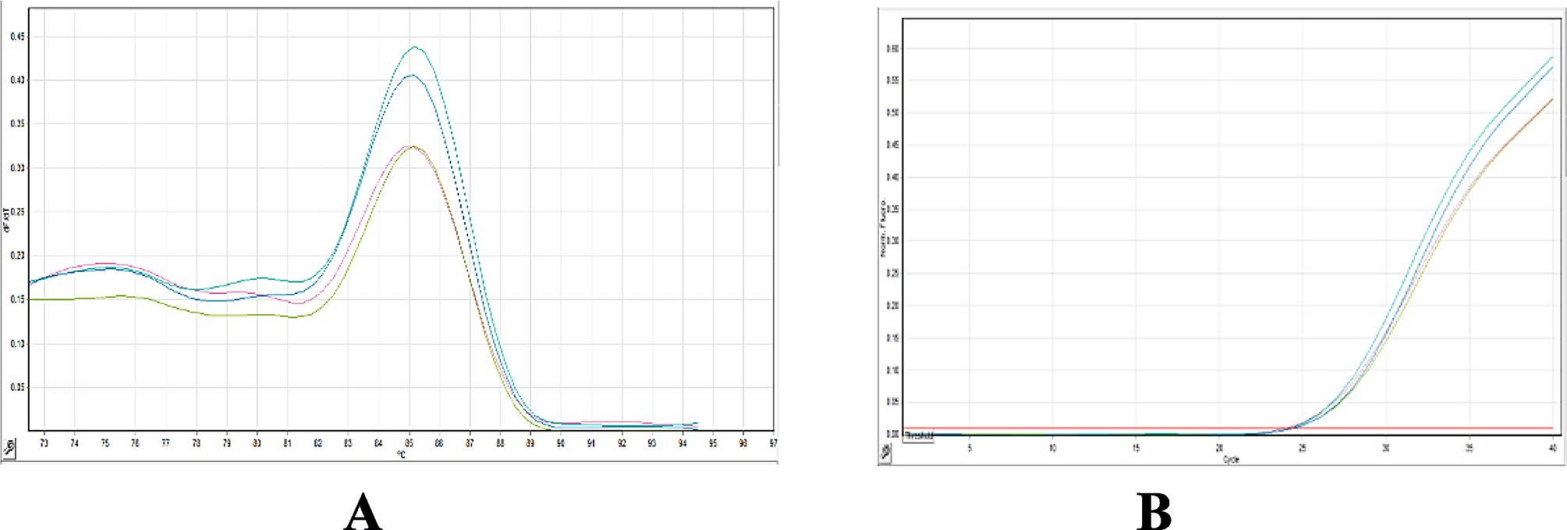
Representative melt curve (**A**) and amplification plot (**B**) from the clinical cohort (*n* = 360; 180 PCa and 180 BPH), illustrating quantitative PCR (qPCR) results following methylation-sensitive restriction enzyme PCR (MSRE-PCR) targeting a specific CpG site in the *SPDEF* promoter region. **A-** Melt curve analysis demonstrates specific amplification of the target amplicon. No melting peaks are observed in non-template control (NTC) wells, confirming the absence of contamination. **B-**Amplification plot shows efficient and specific amplification of target fragments in representative prostate cancer (PCa) samples following enzymatic digestion. These results confirm the sensitivity and specificity of the MSRE-qPCR assay for detecting *SPDEF* promoter methylation in clinical prostate samples

Subsequently, qPCR was performed on the same digested and undigested DNA samples to validate and quantify methylation levels. Figure 8A displays a representative melt curve, while Fig. 8B shows the corresponding amplification plot, confirming specificity of the assay.

In MSRE-PCR, the digested DNA of PCa samples exhibited markedly reduced band intensity compared to their undigested counterparts, indicating hypomethylation at the enzyme recognition site. In contrast, BPH samples retained stronger bands post-digestion, consistent with hypermethylation. Densitometric analysis revealed a significantly lower mean digestion-to-control (D/C) ratio in PCa samples (0.21 ± 0.05) than in BPH (0.83 ± 0.09; *p* ≤ 0.0001).

qPCR analysis further validated these results. Lower ΔCt values (Ct_digested– Ct_undigested) in PCa samples indicated greater accessibility of unmethylated DNA to enzymatic cleavage. The calculated mean hypomethylation level was 92% in PCa versus 15.5% in BPH, consistent across replicate samples. Amplification and melting curve profiles confirmed assay specificity, with non-template controls (NTCs) yielding no signal (Fig. 8).

**Fig. 9:**
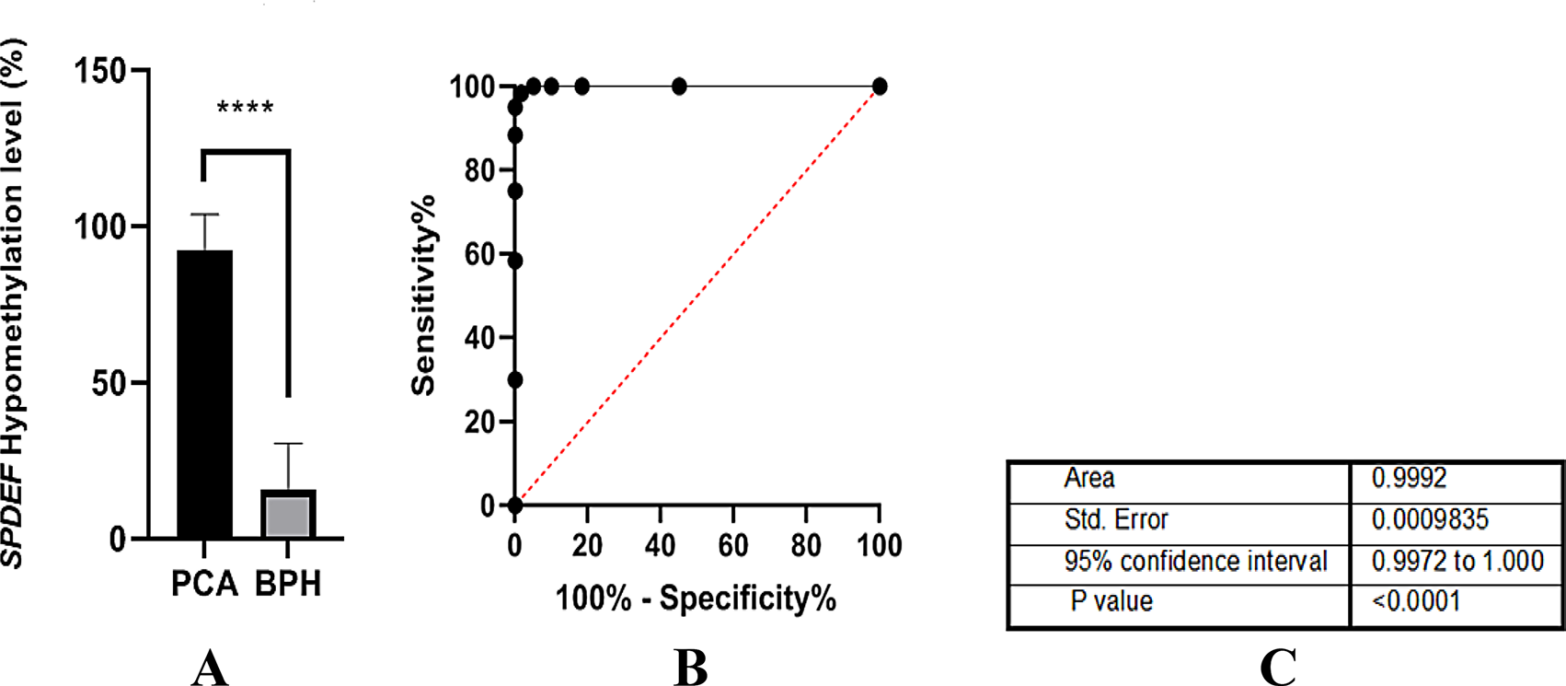
Evaluation of *SPDEF* promoter hypomethylation for prostate cancer (PCa) diagnosis using MSRE-qPCR in clinical samples (*n* = 360; 180 PCa and 180 BPH). A-Quantitative comparison of *SPDEF* hypomethylation levels between PCa and BPH samples. A significantly lower level of *SPDEF* promoter methylation was observed in PCa samples compared to BPH controls (****P < 0.0001).B-Receiver operating characteristic (ROC) curve illustrating the diagnostic performance of *SPDEF* hypomethylation in distinguishing PCa from BPH. The curve demonstrates high sensitivity and specificity, with each plotted dot representing a selected threshold cutoff. C- Summary table of ROC curve parameters: area under the curve (AUC), standard error, 95% confidence interval (CI), and P value. The analysis yielded an AUC of 0.9992, sensitivity of 98.3%, specificity of 98.3%, and a P value < 0.0001, indicating excellent discriminative ability

Both MSRE-PCR and qPCR methods showed high concordance (*R²* = 0.91), underscoring the robustness and reproducibility of the *SPDEF* methylation assay. Using a < 55% methylation threshold, ROC curve analysis (Fig. 9B) showed that *SPDEF* hypomethylation effectively discriminated prostate cancer (PCa) from benign prostatic hyperplasia (BPH) with 98.3% sensitivity and 98.3% specificity (*p* ≤ 0.0001).

To further evaluate its diagnostic performance, we quantified *SPDEF* promoter hypomethylation in the clinical cohort using MSRE-qPCR. Figure 9A illustrates a quantitative comparison of methylation levels between PCa and BPH samples, revealing a significantly lower methylation level in PCa (**** *p* < 0.0001).

Figure 9C summarizes the ROC curve parameters, showing an area under the curve (AUC) of 0.9992, with a standard error of 0.0009835 and a 95% confidence interval ranging from 0.9972 to 1.000. The plotted dots on the ROC curve represent selected threshold cutoffs across the sample distribution.

These results highlight the high diagnostic sensitivity and specificity of *SPDEF* hypomethylation, supporting its utility as a non-invasive epigenetic biomarker to distinguish PCa from BPH in clinical samples.

Table 2 summarizes the MSRE-PCR and qPCR performance metrics, while Table 3 presents correlations between *SPDEF* hypomethylation and clinicopathological variables, including tumor stage (TS), ISUP grade, prostate weight, and age. Significant positive correlations with both TS and grade support the potential prognostic relevance of *SPDEF* promoter CpG site in prostate cancer.

**Table 2:** Summary of comparative *SPDEF* CpG dinucleotide methylation results obtained by MSRE-PCR and qPCR. Includes digestion/control (D/C) ratios, mean ΔCt values, calculated hypomethylation percentages, and method concordance (R² = 0.91) across PCa and BPH samples

Method	Metric	PCa Group	BPH Group	*p*-value
MSRE- PCR	D/C Band Intensity Ratio	0.21 ± 0.05	0.83 ± 0.09	≤ 0.0001
qPCR	ΔCt (Ct_dig– Ct_undig)	Lower ΔCt (indicating **lower methylation**)	Higher ΔCt (indicating higher methylation)	≤ 0.0001
qPCR	Calculated Hypomethylation (%)	92%	15.5%	≤ 0.0001
Correlation	R² (MSRE-PCR vs. qPCR agreement)	0.91	0.91	—

**Table 3:** Correlation between *SPDEF* CpG dinucleotide hypomethylation and clinicopathological parameters in PCa patients (*n* = 180). Assessed variables include tumor stage (TS), Gleason grade (ISUP), prostate weight, and age. Significant positive correlations were observed between hypomethylation levels and both tumor stage and grade

Clinical and Personal Characteristics	PCa (*n* = 180)	SPDEF Gene Hypomethylation (%) Mean±	*p*-Value
Age			≤0.42
43-50	53	77.2%	
50–70	88	60.58%	
70-95	39	66.77%	
**Weight_of_prostate**			**≤0.52**
4–50	100	61.25%	
50–90	40	50.90%	
90–130	40	56.17%	
**Grade (ISUP)**			**≤0.0001**
1	15	5.66%	
2	20	30%	
3	46	55.2%	
4	20	74.%	
5	79	100%	
**TS**			**≤0.0001**
I	7	10%	
II	15	35%	
III	33	64.5%	
IV	70	96%	
V	55	100%	

Abbreviations: *SPDEF* (SAM-pointed domain-containing ETS transcription factor); PCa, prostate cancer; TS, tumor stage; ISUP, International Society of Urological Pathologists

NTC refers to the non-template control (no DNA), confirming the absence of contamination. “Ladder” indicates a 100 bp DNA ladder. C1 and C2 represent non-digested DNA samples (no enzyme), serving as positive amplification controls. D1 and D2 are the corresponding digested DNA samples treated with the HaeIII methylation-sensitive restriction enzyme. The presence of bands in D1/D2 lanes indicates hypomethylation at the target site, as unmethylated DNA is cleaved and remains amplifiable.

- C1/C2: Non-digested controls (PCR without enzyme; amplification confirms DNA integrity).
- D1/D2: Digested samples (PCR with enzyme; amplification only if the region is unmethylated).
- NTC ≠ C1/C2, as NTC lacks DNA entirely, whereas C1/C2 contain intact, undigested DNA.

Left panel: sample with 70% hypomethylation (tumor stage 3, grade 3).

Right panel: sample with 90% hypomethylation (tumor stage 4, grade 4).

### Correlation of *SPDEF* hypomethylation with clinicopathological parameters

*SPDEF* CpG hypomethylation levels were significantly associated with both tumor stage (TS) and histological grade (ISUP) in prostate cancer patients (*n* = 180). Group-wise analysis using the Kruskal–Wallis test followed by Dunn’s multiple comparisons revealed a progressive increase in hypomethylation with advancing tumor stage and grade (Fig. 2A and B).

Statistically significant differences were observed between most consecutive TS and ISUP groups, with the highest hypomethylation levels detected in TS-4 and G5 (*P* < 0.0001).

To further validate these findings, linear regression analysis confirmed a strong positive correlation between *SPDEF* hypomethylation and both tumor stage (*r* = 0.9574) and ISUP grade (*r* = 0.9348), with narrow confidence intervals and highly significant *P* values (< 0.0001; Fig. 2C and D).

These findings underscore the potential utility of *SPDEF* hypomethylation as an epigenetic biomarker indicative of disease progression and tumor aggressiveness in prostate cancer [25].

### Correlation between PSA levels and *SPDEF* promoter methylation

To evaluate the relationship between *SPDEF* promoter methylation and serum PSA levels, Spearman’s rank correlation analysis was performed using methylation values derived from both PCR-based detection methods. PSA values were treated as continuous variables across the full range (0–120 ng/mL). Spearman’s rank correlation revealed a moderate, statistically significant negative correlation between PSA levels and methylation percentages obtained via MSRE-PCR (ρ = − 0.364, *p* = 1.25 × 10⁻⁸). Similarly, qPCR-derived methylation data showed a comparable inverse correlation (ρ = − 0.341, *p* = 1.10 × 10⁻⁷). These findings suggest that increasing PSA levels are associated with decreasing methylation of the *SPDEF* promoter, as measured by both PCR methods (Fig. 3).

## Discussion

Globally, approximately one million new cases of prostate cancer (PCa) are diagnosed annually, with more than 300,000 resulting in death. Early detection is critical to reducing PCa-related morbidity and mortality, especially considering the impact of genetic diversity and environmental factors across different age and ethnic groups, which contribute to disparities in incidence and outcomes [26, 27]. Implementing a screening program based on DNA methylation biomarkers could significantly enhance cancer diagnosis and management worldwide [28].

To investigate the potential role of the *SPDEF* gene in PCa, we assessed the methylation status of *SPDEF* in 360 peripheral blood samples (180 PCa and 180 benign prostatic hyperplasia [BPH] controls) using methylation-sensitive restriction enzyme PCR (MSRE-PCR) and quantitative PCR (qPCR). We then analyzed the association between *SPDEF* methylation and clinical parameters, including tumor stage (TS), Gleason grade (ISUP), and total prostate-specific antigen (tPSA) levels.SPDEF (prostate-derived ETS factor) has been implicated in tumor biology; however, its precise function in cancer remains debated [29, 30]. Aberrant methylation of *SPDEF* has been reported in PCa [31]. Given its role in restricting cellular plasticity and promoting a luminal epithelial phenotype, *SPDEF* is considered critical in tumor growth and metastasis [32].

Bioinformatics analysis using TCGA and GEO datasets supported our experimental results, showing that *SPDEF* expression was significantly elevated in PCa tissues, coinciding with CpG site hypomethylation in the promoter region (cg11346722, chr6:34544344–34544482). These data underscore the epigenetic regulation of *SPDEF* and its potential as a PCa biomarker. Other studies have similarly reported SPDEF overexpression in cancers including prostate cancer [31, 33].

However, in this study, a strong positive two-way correlation was observed between *SPDEF* hypomethylation and tumor grade (*r* = 0.947) and stage (*r* = 0.957) in a linear regression model. A comparison of TS levels based on *SPDEF* hypomethylation levels indicated that the rate of *SPDEF* hypomethylation was significantly higher in TS 4 and 3 than in TS 1 and 2 (*p* ≤ 0.001). This discrepancy between T4 and T1 was considerably different (*p* ≤ 0.0001), indicating a straightforward correlation between lower methylation levels of *SPDEF* and the metastatic phase of PCa. While PCa in the indolent phase (T1, T2) had lower levels of hypomethylation, this comparison was also adapted to the International Society of Urological Pathologists (ISUP) level alterations. This suggests that an increase in *SPDEF* gene hypomethylation is directly linked to cellular alterations in prostate tissue cells and their carcinogenic development and that the change in *SPDEF* hypomethylation after TS2 abruptly exhibited a significant increase. This problem was also observed in G2 and G3, indicating that these are critical junctures for methylation alterations at the molecular level, as evidenced by the correlation between *SPDEF* expression and ISUP in PCa clinical samples [12]. Importantly, Kruskal–Wallis tests with Dunn’s multiple comparisons confirmed statistically significant differences in *SPDEF* CpG hypomethylation across tumor stages (TS1–TS4) and ISUP grades (G1–G5), with the highest hypomethylation levels observed in TS-4 and G5 (*P* < 0.0001). Spearman’s rank correlation analysis revealed strong positive correlations between hypomethylation and tumor stage (ρ = 0.9574) and ISUP grade (ρ = 0.9348), both highly significant (*P* < 0.0001). In addition, receiver operating characteristic (ROC) curve analysis showed that *SPDEF* promoter hypomethylation distinguished PCa from BPH with an AUC of 0.9992, 98.3% sensitivity, and 98.3% specificity (*P* < 0.0001), underscoring its diagnostic value. These results support *SPDEF* methylation as a highly accurate, noninvasive biomarker for both diagnosis and clinical staging of prostate cancer.This supports the potential of DNA methylation patterns in distinguishing PCa from BPH through minimally invasive blood-based assays [34].

Real-time qPCR results supported these findings, showing that 99/180 (55%) PCa samples exhibited complete hypomethylation, compared with 3/180 BPH and 15/180 healthy samples. Consistent with prior studies showing hypomethylation of *SPDEF* in LNCaP prostate cancer cells [7], our data point to significant epigenetic dysregulation of *SPDEF* in PCa. Notably, our use of MSRE-PCR and qPCR provided a sensitive, noninvasive, and cost-effective method to assess methylation in blood-derived DNA [35].

Recent studies have provided evidence supporting this statement. Specifically, research has demonstrated that *SPDEF* expression is inversely correlated with methylation at CpG dinucleotides within its gene in prostate cancer tissues. Higher methylation levels are associated with decreased *SPDEF* expression, particularly in advanced stages of the disease. Furthermore, treatment with DNA methyltransferase inhibitors, such as 5-Aza-dC, has been shown to restore *SPDEF* expression and reduce the invasive properties of prostate cancer cells [7].

Additionally, another study reported that both enhancer and promoter regions of the *SPDEF* gene undergo significant hypermethylation in aggressive prostate cancer cell models. This epigenetic modification contributes to the suppression of *SPDEF* expression, suggesting a role in metastasis and therapeutic resistance [13]. These findings underscore the importance of epigenetic mechanisms in regulating *SPDEF* expression and their potential impact on prostate cancer progression.

The target region within the *SPDEF* promoter (chr6:34544344–34544482, cg11346722) emerges as a promising epigenetic biomarker for PCa. This study represents the first to evaluate *SPDEF* methylation as a noninvasive, cost-effective diagnostic marker in blood-derived DNA. Region-specific hypomethylation is a common feature in tumor genomes, and methylation levels in peripheral leukocytes have previously been linked to cancer risk and progression [36, 37].

*SPDEF* did not accelerate the growth of tumors in low *SPDEF*-expressing cancers with basal-like differentiation but accelerated tumor growth in high *SPDEF*-expressing tumors with classical differentiation [38].

In this study, PCa samples were compared to BPH samples using experimental assays and analytics to identify potential epigenetic biomarkers for PCa. Bioinformatic analysis has been extensively used in cancer research [29]. It has been revealed that *SPDEF* has both oncogenic and tumor-suppressive properties [38].

It has been demonstrated that the sensitivity of diagnosing prostate cancer can be increased by utilizing a panel of CpG dinucleotide methylation markers in conjunction with routine histological assessment of needle biopsies [39].

No significant correlation was found among patient age, prostate weight, and *SPDEF* hypomethylation in patients with PCa. The function of *SPDEF* in tumor metastasis in vivo in any system has not been investigated to date [30]. *SPDEF* gene has been shown to have both pro- oncogenic properties, and these actions vary depending on the stage [11].

Survival analysis at the cg11346722 site using the SMART tool revealed that patients with hypomethylation of this locus had improved overall survival (Supplementary S5 _Fig S1). This observation is consistent with literature indicating that tumors with higher CD8 + T-cell infiltration often exhibit better prognosis and disease-free survival [11]. To further investigate this, we utilized the TIMER2.0 database to examine the relationship between *SPDEF* expression and immune infiltration profiles in prostate cancer. The results indicated a significant positive correlation between *SPDEF* expression and CD8 + T-cell infiltration (Supplementary S6 _Fig S1). These findings suggest that hypomethylation-driven upregulation of *SPDEF* may influence the tumor immune microenvironment, potentially through modulation of immune cell recruitment or function. However, we emphasize that this conclusion is based solely on bioinformatic analyses, and future experimental studies are warranted to validate these observations.

Notably, the most important clinical requirement in prostate cancer is metrics that are more repeatable and dependable than the ones that are now in use. The best proven predictive metric, the Gleason grade, has a significant inter-observer variability of up to 40% in individual biopsies [40].

Importantly, our correlation analysis was performed using PSA as a continuous variable rather than stratifying within the 4–10 ng/mL gray zone. This approach is statistically more robust and avoids the limitations of arbitrary subgrouping, which may dilute the interpretability of biomarker relationships in heterogeneous clinical populations. The consistency of the negative correlation across two independent methylation detection platforms reinforces the potential of *SPDEF* methylation as a clinically informative epigenetic biomarker in prostate cancer. Studies have highlighted that treating PSA as a continuous variable can enhance the predictive performance of diagnostic models, especially in the gray-zone range [41].

Future studies with larger cohorts and longitudinal PSA tracking may further clarify the clinical utility of *SPDEF* methylation in early detection and risk stratification. The role of *SPDEF* as a co-activator that induces the expression of prostate-specific antigen (PSA) in LNCaP prostate tumors has been established [42].

Using MSRE-PCR and qPCR, we detected a significant decrease in the methylation (hypomethylation) of CpG dinucleotides in *SPDEF* in DNA extracted from peripheral blood samples. Methylation-sensitive restriction enzymes (MSREs), including CpG dinucleotides at their recognition islands, can be used to evaluate models of DNA methylation alteration in human diagnostics. Only unmethylated sections can be broken down [43, 44], whereas methylated sections remain intact, as 5 mC suppresses the activities of these enzymes. For efficient digestion and amplification, only methylated DNA must yield detectable PCR results [45].

Methylation testing in sizable cohort studies is made possible by a combination of MSRE digestion and PCR-based detection, enabling the use of high-throughput PCR technologies. The detection of methylated *SPDEF* and quantification of methylated DNA in individual samples are made possible by the simple and sophisticated design of MSRE-PCR assays [46, 47].

The clinical relevance of *SPDEF* methylation is further supported by prior studies using MSRE-qPCR in other cancers (e.g., *ITGA6* in breast cancer, *DOK7* in gastric cancer) [48, 49]. However, our study represents the first comprehensive assessment of *SPDEF* methylation in peripheral blood for PCa detection and staging.

Prior studies have demonstrated that multi-marker methylation panels may enhance sensitivity and specificity for PCa detection. For instance, a study combining *APC*,* GSTP1*, and *RARβ2* methylation markers achieved 100% sensitivity and specificity in tissue samples, and high diagnostic accuracy in urine [50].

Another study reported 100% sensitivity and 97% specificity for a panel detecting PCa in histologically benign cores [51]. Similarly, a panel of *APC*,* GSTP1*, and *IGFBP3* differentiated PCa from BPH with 91% sensitivity and 85.9% specificity [52]. These findings support integrating *SPDEF* methylation into broader diagnostic panels.

A limitation of this study is the sample size, which may affect the generalizability of the findings. Future studies involving larger and more diverse populations are warranted to validate these results and to explore the mechanistic role of *SPDEF* in tumor progression and immune modulation.

## Conclusion

This study presents the first comprehensive evaluation of *SPDEF* gene CpG dinucleotide methylation as a potential epigenetic biomarker associated with prostate cancer (PCa) progression, defined by tumor stage and histological grade. Genomic DNA was extracted from blood leukocytes of patients with PCa and individuals with benign prostatic hyperplasia (BPH), the latter serving as the control group. Methylation status was assessed using a combination of methylation-sensitive restriction enzyme PCR (MSRE-PCR) and quantitative PCR (qPCR).

A significant inverse relationship was observed between *SPDEF* methylation levels and PCa severity. Early-stage tumors with low Gleason grade exhibited a hypermethylated *SPDEF* profile, whereas advanced-stage and high-grade tumors demonstrated pronounced *SPDEF* hypomethylation. Notably, BPH samples consistently retained a hypermethylated *SPDEF* pattern, underscoring the biomarker’s discriminatory potential.

These findings suggest that *SPDEF* CpG dinucleotide methylation status may serve as a noninvasive, blood-based biomarker for PCa diagnosis and disease monitoring. Further validation in larger, multi-center cohorts is warranted to confirm its clinical applicability and integration into routine prostate cancer screening and prognostic workflows.

A figure to better understanding about of the possible mechanisms related to hypomethylation in Prostate cancer.

https://clinicalepigeneticsjournal.biomedcentral.com/articles/10.1186/s13148-021-01111-8.

## Electronic supplementary material

Below is the link to the electronic supplementary material.

Supplementary Material 1

Supplementary Material 2

Supplementary Material 3

Supplementary Material 4

Supplementary Material 5

Supplementary Material 6

Supplementary Material 7

## Data Availability

The data generated or analyzed during the study are available upon request for ethical, legal, or commercial reasons. The corresponding author, Dr. Seyed Ahmad Aleyasin, should be contacted at (mailto: sogand@nigeb.ac.ir).

## Abbreviations

SPDEF: SAM-pointed domain-containing ETS transcription factor
PCa: Prostate cancer
BPH: Benign prostatic hyperplasia
NC: Normal control
TS: tumor stage
RE: Restriction enzymes
BLs: Blood leukocytes
NTC: Non-treated control
PSA: prostate specific antigen
tPSA: Total PSA
