# Supplementary material for: CpG dinucleotide methylation of the SPDEF gene as a blood-based epigenetic biomarker for prostate cancer diagnosis
Source: BMC Urol. 2025 Jun 2;25:145. doi: 10.1186/s12894-025-01824-5 (PMC12128380; doi:10.1186/s12894-025-01824-5)
Supplement: Supplementary file 1 — Supplementary Material 1 [file 12894_2025_1824_MOESM1_ESM.docx]

**Supplementary1_1: Chromosomal Localization of Differentially Methylated Genes in Prostate Cancer**

A circular ideogram map was generated to visualize the chromosomal distribution of differentially methylated genes identified in the GEO datasets GSE26126 and GSE15727. The figure highlights the genomic loci of overlapping differentially methylated regions (DMRs) common to both datasets.

As shown in Supplementary Figure 1, *SPDEF* is localized on chromosome 6p21.31, marked in red, indicating its significant hypomethylation in prostate cancer tissues compared to benign counterparts. Other hypermethylated and hypomethylated genes are mapped across different chromosomes, reflecting the widespread epigenetic reprogramming characteristic of prostate cancer.


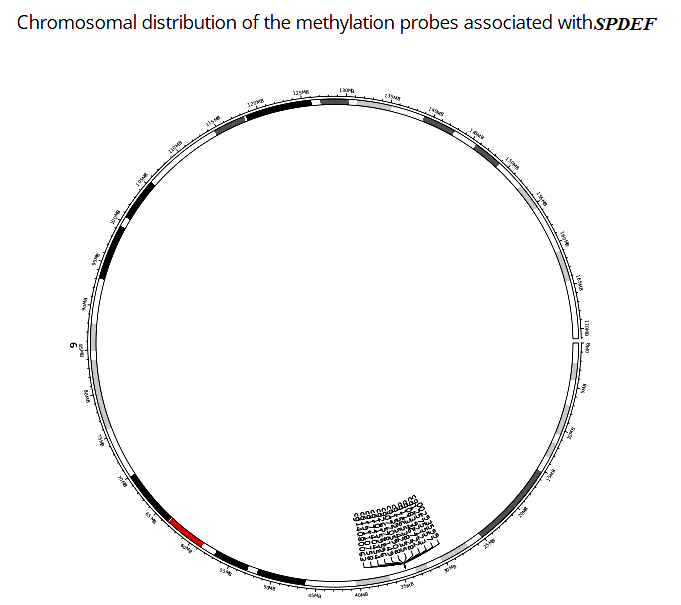


**Supplementary Figure 1.** **Chromosomal distribution of the methylation probes associated with *SPDEF*.**
This circular plot illustrates the distribution of methylation probes targeting the *SPDEF* gene on chromosome 6. The highlighted region in red indicates the location of the specific CpG site (cg11346722) in the *SPDEF* promoter region, which was identified as significantly hypomethylated in prostate cancer (PCa) samples. Other associated CpG sites, in varying shades, are also shown across the chromosome for reference.. <http://www.bioinfo-zs.com/smartapp/#tab-4008-2>

**Supplementary 1-2: Chromosomal Distribution of Hypomethylated CpG Sites in Prostate Cancer**

Genome-wide mapping of hypomethylated CpG sites was performed across all autosomes and sex chromosomes using merged methylation data from GSE26126 and GSE15727. This lollipop-style karyogram depicts the density and distribution of 2,584 significantly hypomethylated CpG sites along individual chromosomes.

Each vertical bar represents a hypomethylated CpG locus, with red bars highlighting genes of interest, including *SPDEF*, which is located on chromosome 6 and showed consistent hypomethylation in prostate cancer tissues. The plot demonstrates a broad, uneven distribution of hypomethylation events across the genome, reflecting complex epigenetic deregulation in prostate cancer.


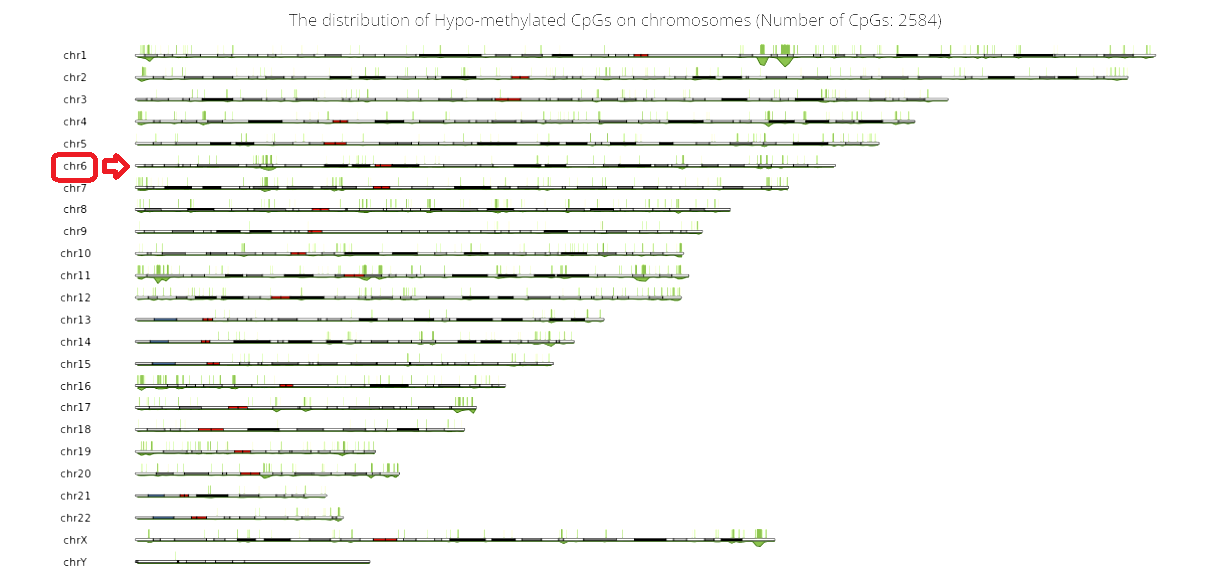


**Supplementary Figure 1-2.** Karyotype-style visualization of 2,584 hypomethylated CpG sites distributed across all human chromosomes. Red peaks indicate key genes identified in overlapping datasets, including *SPDEF*. <http://www.bioinfo-zs.com/smartapp/#tab-4008-2>
