# Supplementary material for: CpG dinucleotide methylation of the SPDEF gene as a blood-based epigenetic biomarker for prostate cancer diagnosis
Source: BMC Urol. 2025 Jun 2;25:145. doi: 10.1186/s12894-025-01824-5 (PMC12128380; doi:10.1186/s12894-025-01824-5)
Supplement: Supplementary file 2 — Supplementary Material 2 [file 12894_2025_1824_MOESM2_ESM.docx]

**Supplementary 2 _Table-1. The methylation-sensitive restriction enzyme PCR (MSRE-PCR) conditions for *SPDEF* promoter.**

| **Qualitative PCR conditions** | | **Temperature (°C)** | **Time** |
| --- | --- | --- | --- |
| Initial denaturation | | 90 | 10 minutes |
| 40 cycles | Denaturation | 95 | 45 seconds |
|  | Annealing | 72 | 45 seconds |
|  | Extension | 66 | 30 seconds |
| Final extension | | 80 | 7 minutes |

**Supplementary 2 _Table-2. The quantitative PCR (qPCR) conditions for SPDEF promoter.**

| **Quantitative PCR conditions** | | **Temperature (°C)** | **Time** |
| --- | --- | --- | --- |
| Initial denaturation | | 91 | 4 minutes |
| 45 cycles | Denaturation | 93 | 44 seconds |
|  | Annealing | 65 | 60 seconds |
|  | Extension | 70 | 60 seconds |
| Final extension | | 77 | 5 minutes |

**Supplementary 2 _Table-3**.**The *SPDEF* gene abbreviation, forward and reverse primer sequences, product size, restriction enzyme , and recognition site.**

**Gene Primer Sequence (5′ ⟶ 3′) Product Size Enzyme Recognition Site**

***SPDEF* F: GACCCACTCGACGTATCTCT 176 bp HaeIII 5′...GG^CC... 3′**

**R: ACCTCAGACCACAGGCAGGC 3′...CC^GG... 5′**
