# Supplementary material for: CpG dinucleotide methylation of the SPDEF gene as a blood-based epigenetic biomarker for prostate cancer diagnosis
Source: BMC Urol. 2025 Jun 2;25:145. doi: 10.1186/s12894-025-01824-5 (PMC12128380; doi:10.1186/s12894-025-01824-5)
Supplement: Supplementary file 3 — Supplementary Material 3 [file 12894_2025_1824_MOESM3_ESM.docx]

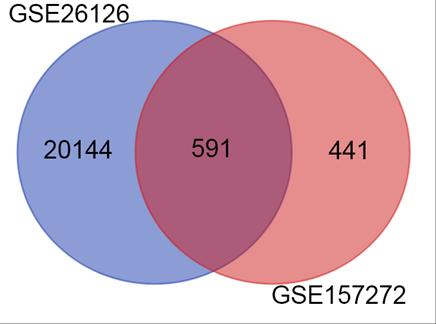


**Supplementary 3 _Fig.1** Venn diagram showing the overlap of hypomethylated genes identified in two independent prostate cancer datasets, both analyzed using the Illumina Infinium HumanMethylation27 BeadChip platform. Differential methylation analysis was performed using the GEO2R tool (<https://www.ncbi.nlm.nih.gov/geo/geo2r/>).
