# Supplementary material for: CpG dinucleotide methylation of the SPDEF gene as a blood-based epigenetic biomarker for prostate cancer diagnosis
Source: BMC Urol. 2025 Jun 2;25:145. doi: 10.1186/s12894-025-01824-5 (PMC12128380; doi:10.1186/s12894-025-01824-5)
Supplement: Supplementary file 5 — Supplementary Material 5 [file 12894_2025_1824_MOESM5_ESM.docx]

**Supplementary 4 Methylation Profiling of *SPDEF*-Associated CpG Sites in Prostate Adenocarcinoma**

To further investigate *SPDEF* promoter methylation in prostate cancer, we examined the β-values of 13 CpG sites annotated to the *SPDEF* gene using the UALCAN database (<http://ualcan.path.uab.edu/>). Methylation levels were compared between normal and tumor samples in The Cancer Genome Atlas (TCGA) prostate adenocarcinoma (PRAD) cohort.As shown in Supplementary Figures 4, most CpG sites exhibited significant hypomethylation in tumor tissues compared to normal prostate tissues (Wilcoxon p-values < 0.05). Notably, **cg11346722** (highlighted in yellow) showed one of the most pronounced differences in methylation, supporting its selection for further targeted validation using MSRE-PCR and qPCR assays.The aggregation panel at the bottom-left corner illustrates overall *SPDEF* methylation trends across all CpG sites. These data support a consistent reduction in *SPDEF* methylation in prostate tumors, reinforcing its relevance as a potential diagnostic biomarker.


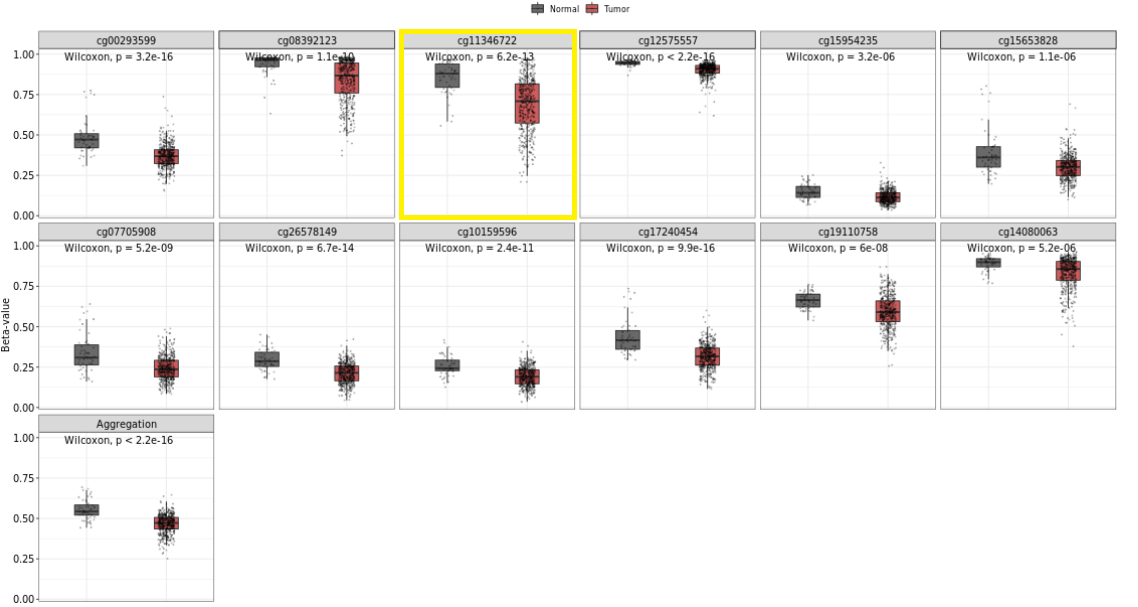


**Supplementary 4 _Fig.1** Boxplots showing methylation β-values for 13 *SPDEF*-associated CpG sites in TCGA-PRAD. Tumor samples (red) are compared to normal controls (gray). The CpG site cg11346722, selected for downstream analysis, is highlighted with a yellow border. Aggregated methylation levels across all probes are shown in the bottom-left panel. <http://www.bioinfo-zs.com/smartapp/#tab-4008-2>


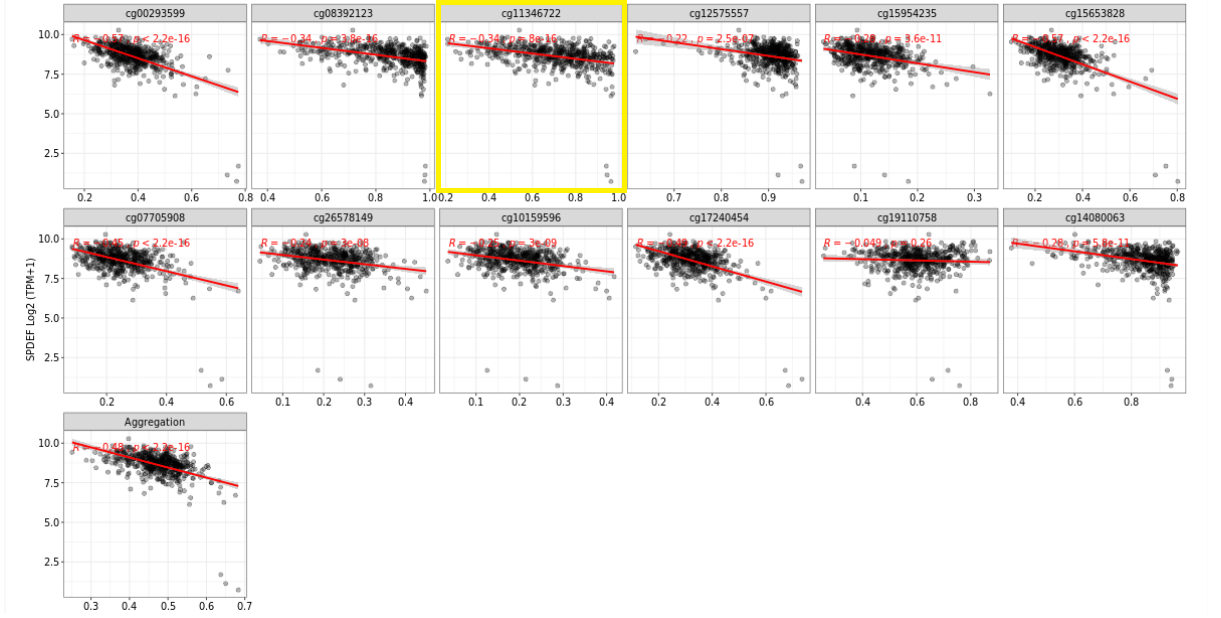


**Supplementary 4_Fig. 2** The important discovery is that *SPDEF* expression and the methylation state of the CpG island cg11346722 site have a negative significant correlation (Pearson R = -0.34, p = 3.8e.16). In prostate adenocarcinoma (PRAD), there is a Pearson link between DNA methylation (M value) and expression (*SPDEF*). A gene-level correlation demonstrates a substantial negative relationship between the methylation of cg11346722, and the expression of *SPDEF*. <http://www.bioinfo-zs.com/smartapp/#tab-4008-2>
