# Supplementary material for: CpG dinucleotide methylation of the SPDEF gene as a blood-based epigenetic biomarker for prostate cancer diagnosis
Source: BMC Urol. 2025 Jun 2;25:145. doi: 10.1186/s12894-025-01824-5 (PMC12128380; doi:10.1186/s12894-025-01824-5)
Supplement: Supplementary file 6 — Supplementary Material 6 [file 12894_2025_1824_MOESM6_ESM.docx]

**Supplementary 5 Association Between SPDEF Expression and Overall Survival in Prostate Cancer**

To evaluate the prognostic significance of SPDEF expression in prostate cancer, Kaplan–Meier survival analysis was performed using TCGA-PRAD data via the GEPIA2 online tool (<http://gepia2.cancer-pku.cn/>). Patients were stratified into high and low SPDEF expression groups based on the median expression value (n = 248 each).As shown in Supplementary Figure 5, no significant difference in overall survival (OS) was observed between the high and low SPDEF expression groups (p = 0.988). The hazard ratio (HR) was 1.01, with a 95% confidence interval (CI) ranging from 0.28 to 3.58. These results suggest that SPDEF expression does not have a significant prognostic impact on overall survival in prostate adenocarcinoma.


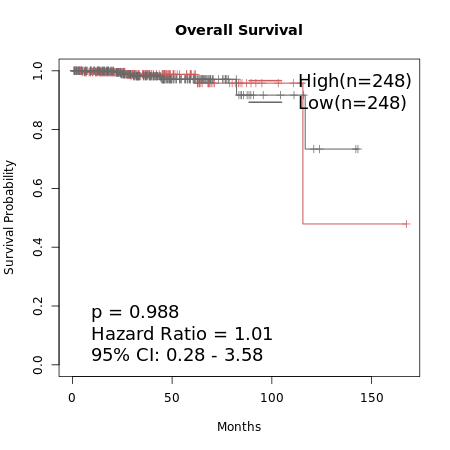


**Supplementary 5_Fig.1** Kaplan–Meier overall survival curve for prostate adenocarcinoma patients stratified by SPDEF expression level. No statistically significant difference in survival was found between the high and low SPDEF expression groups (p = 0.988; HR = 1.01; 95% CI: 0.28–3.58).<http://www.bioinfo-zs.com/smartapp/>
