# Supplementary material for: CpG dinucleotide methylation of the SPDEF gene as a blood-based epigenetic biomarker for prostate cancer diagnosis
Source: BMC Urol. 2025 Jun 2;25:145. doi: 10.1186/s12894-025-01824-5 (PMC12128380; doi:10.1186/s12894-025-01824-5)
Supplement: Supplementary file 7 — Supplementary Material 7 [file 12894_2025_1824_MOESM7_ESM.docx]

**Supplementary 6. Correlation Between *SPDEF* Expression and Immune Cell Infiltration in Prostate Adenocarcinoma**

To investigate the potential relationship between *SPDEF* expression and the tumor immune microenvironment, we assessed the correlation between *SPDEF* mRNA expression and immune infiltration levels in prostate adenocarcinoma (PRAD) using the TIMER database (<http://timer.cistrome.org/>). The TIMER algorithm estimates immune cell infiltration levels from bulk RNA-seq data across TCGA cancer types.

As shown in Supplementary Figure 6, a statistically significant positive correlation was observed between *SPDEF* expression and tumor purity (Spearman’s ρ = 0.20, *p* = 3.78 × 10⁻⁵), indicating that higher *SPDEF* levels are associated with increased tumor cell content in prostate tumors. Additionally, *SPDEF* expression showed a significant positive correlation with CD8⁺ T cell infiltration (Spearman’s ρ = 0.206, *p* = 2.32 × 10⁻⁵). These results suggest that *SPDEF* may play a role in modulating immune infiltration, particularly CD8⁺ T cell presence, within the prostate tumor microenvironment.


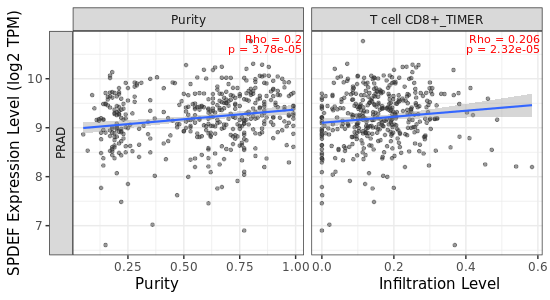


**Supplementary 6 _Fig 1.** **Correlation of *SPDEF* gene expression with tumor purity and CD8⁺ T cell infiltration in prostate adenocarcinoma (PRAD).**
Scatter plots depict the relationship between *SPDEF* expression (log₂ TPM) and (left) tumor purity and (right) CD8⁺ T cell infiltration level in PRAD (TCGA cohort), derived using the TIMER database. Blue lines indicate fitted regression curves with confidence intervals. Spearman’s ρ and associated *p*-values are shown in red.
